# Furanoid F-Acid F6 Uniquely Induces NETosis Compared to C16 and C18 Fatty Acids in Human Neutrophils

**DOI:** 10.3390/biom8040144

**Published:** 2018-11-13

**Authors:** Meraj A. Khan, Cecil Pace-Asciak, Jassim M. Al-Hassan, Mohammad Afzal, Yuan Fang Liu, Sosamma Oommen, Bincy M. Paul, Divya Nair, Nades Palaniyar

**Affiliations:** 1 Program in Translational Medicine, Peter Gilgan Centre for Research and Learning, The Hospital for Sick Children, Toronto, ON M5G 0A4, Canada; meraj.khan@sickkids.ca (M.A.K.); cecil.pace-asciak@sickkids.ca (C.P.-A.); yuanfang.liu@sickkids.ca (Y.F.L.); 2 Department of Pharmacology, University of Toronto, Toronto, ON M5S 1A8, Canada; 3 Department of Biological Sciences, Faculty of Science, Kuwait University, Safat 13060, Kuwait; jassim5577_@hotmail.com (J.M.A.-H.); m.afzal@ku.edu.kw (M.A.); bincymaniyalil@gmail.com (B.M.P.); divyajs2002@yahoo.co.in (D.N.); 4 Department of Zoology, CMS College, Kottayam 686001, India; sosamma@cmscollege.ac.in; 5 Departments of Lab Medicine and Pathobiology, and Institute of Medical Sciences, Faculty of Medicine, University of Toronto, Toronto, ON M5G 0A4, Canada

**Keywords:** furanoid F-acids (F6), catfish lipids, long-chain fatty acids, NETosis, NADPH oxidase, ROS, MAP kinases, citrullination of histone, transcription

## Abstract

Various biomolecules induce neutrophil extracellular trap (NET) formation or NETosis. However, the effect of fatty acids on NETosis has not been clearly established. In this study, we focused on the NETosis-inducing ability of several lipid molecules. We extracted the lipid molecules present in Arabian Gulf catfish (*Arius bilineatus*, Val) skin gel, which has multiple therapeutic activities. Gas chromatography–mass spectrometry (GC-MS) analysis of the lipid fraction-3 from the gel with NETosis-inducing activity contained fatty acids including a furanoid F-acid (F6; 12,15-epoxy-13,14-dimethyleicosa-12,14-dienoic acid) and common long-chain fatty acids such as palmitic acid (PA; C16:0), palmitoleic acid (PO; C16:1), stearic acid (SA; C18:0), and oleic acid (OA; C18:1). Using pure molecules, we show that all of these fatty acids induce NETosis to different degrees in a dose-dependent fashion. Notably, F6 induces a unique form of NETosis that is rapid and induces reactive oxygen species (ROS) production by both NADPH oxidase (NOX) and mitochondria. F6 also induces citrullination of histone. By contrast, the common fatty acids (PA, PO, SA, and OA) only induce NOX-dependent NETosis. The activation of the kinases such as ERK (extracellular signal-regulated kinase) and JNK (c-Jun N-terminal kinase) is important for long-chain fatty acid-induced NETosis, whereas, in F-acid-induced NETosis, Akt is additionally needed. Nevertheless, NETosis induced by all of these compounds requires the final chromatin decondensation step of transcriptional firing. These findings are useful for understanding F-acid- and other fatty acid-induced NETosis and to establish the active ingredients with therapeutic potential for regulating diseases involving NET formation.

## 1. Introduction

Neutrophils are the major innate immune cells (50–70%) present in the circulation and infiltrate first into the site of infection, injury, or inflammation. Studies conducted in the last decade show that neutrophils form neutrophil extracellular traps (NETs), web-like chromatin structures, decorated with antimicrobial peptides help to ensnare microbial pathogens [1,2]. Several microbial components and cytokines of the host can induce NETosis [3,4,5]. However, f-acids and common fatty acids were not shown to induce NETosis.

We and others have studied the regulatory mechanism of NET formation (NETosis) [6,7,8,9,10,11]. These studies indicate that different agonists induce NETosis by two major pathways, either by NADPH oxidase (NOX)-dependent (mediated by reactive oxygen species (ROS) generated from NOX-induced by agonist such as phorbol 12-myristate 13-acetate (PMA)) and/or NOX-independent pathways (mediated by mitochondrial ROS, calcium influx, activation of different mitogen-activated protein kinases (MAPKs) and citrullination of histone 3 activated by calcium ionophores such as A23187) [6,12,13,14,15,16,17,18,19,20,21]. Our previous studies show that NOX-independent NETosis is rapid (30–60 min) and involves citrullination of histones whereas NOX-dependent NETosis is relatively slow (2–5 h) [6,8,19]. Our recent findings established a unifying model of NETosis: Kinase activation leading to transcriptional firing is essential to decondense chromatin in both NOX-dependent and -independent NETosis [8].

Preparations from gel secreted from the skin of Arabian Gulf catfish (*Arius bilineatus*, Val), have been shown by one of us (JMAH) to possess several therapeutic activities including anti-inflammatory and wound healing properties [22,23,24]. In this study, we focused on identifying important groups of lipid molecules that could induce NETosis as a means of identifying their therapeutic actions. We isolated lipids present in the gel and identified the NETosis pathways regulated by five of the fatty acids present in NETosis-inducing fractions. Interestingly, F-acid F6 induces a form of NETosis, induced by both NOX-dependent and NOX-independent mechanisms whereas certain common long-chain fatty acids induce NETosis only via NOX-dependent mechanism. This new information could be useful for understanding the mechanisms of F6- and fatty acid-induced NETosis, and in formulating potent lipid mixtures for regulating NETosis and inflammation.

## 2. Materials and Methods

### 2.1. Ethics Approval Statement

The study protocol has been approved by the Research Ethics Board (REB) of the Hospital for Sick Children. Methods and procedures were performed in accordance with the REB guidelines and regulations. All the donors signed an informed consent form before participating in the study.

### 2.2. Buffer and Reagent Preparation

SytoxGreen DNA binding dye, DHR123, and MitoSox dye were obtained from Molecular Probes (Thermo Fisher Scientific, Waltham, MA, USA). RPMI 1640 medium (Invitrogen, Carlsbad, CA, USA) supplemented with 10 mM 4-(2-hydroxyethyl)-1-piperazineethanesulfonic acid (HEPES) buffer was used for the *ex vivo* experiments unless otherwise stated. The following compounds were purchased from Sigma (Oakville, ON, Canada): C16:0; C16:1∆9; C18:0; C18:1∆9, and furanoid acid F6 (19C22:6; or 12,15-epoxy-13,14-dimethyleicosa-12,14-dienoic acid) from Cayman Chemical Company (Ann Arbor, MI, USA). Selected doses of all compounds were dissolved in 4 µL ethanol, followed by incubation buffer and mixed just before addition to the incubation wells (100 µL volume). Buffers, pharmacological agonists, inhibitors, and other reagents were purchased from Sigma-Aldrich unless otherwise stated.

### 2.3. Analyses of Lipid Fraction-3 (Ft-3) That Activates NETosis

The lipids from the freeze-dried epidermal catfish secretion were dissolved in sodium methoxide in dry methanol and left overnight at 23 °C. The methanol was then evaporated, water was added, and the mixture was extracted with diethyl ether. The ether extract was dried over sodium sulfate, filtered, and evaporated to dryness in a rotary evaporator. The residue was loaded on a column of silica gel prepared in hexane, and the column was eluted with hexane, hexane:diethyl ether (7:3 *v*/*v*), and finally with hexane:diethyl ether (1:1, *v*/*v*). The fraction labeled Ft-3 was eluted with the 7:3 mixtures.

### 2.4. Gas Chromatography-Mass Spectrometry 

The GC analysis was performed in an Agilent 7890A GC system connected to an Agilent 5975C mass spectrometer operated in the EI (electron impact) mode, column TG-1MS 30 m × 0.25 mm × 0.25 μm—a temperature gradient was employed that started at 70 °C then increased at 10 °C/min to 340 °C and was held for 10 min (37 min total run time). Analysis of Ft-3 was carried out by gas chromatography-mass spectrometry (GC-MS) using the following conditions. Samples were analyzed as the methyl ester trimethylsilyl ether derivatives after the reaction of the methyl esters (see above) in 25 µL TRISILZ (Pierce Chem Co., Rockford, IL, USA) heated for 20 min at 65 °C. Aliquots (1 µL) were injected directly into the GC-MS in splitless mode with the inlet at 250 °C. Helium was used as the carrier gas at a constant flow of 1 mL/min. Compound identification was made through comparison with the NIST Mass Spectral Search Program for the NIST/EPA/NIH Mass Spectral Library Version 2.0 g build 19 May 2011. Positive identity was made by comparison with authentic F6 (Cayman Chemical Co., Ann Arbor, MI, USA).

### 2.5. Human Neutrophil Preparation

At the nursing station of the Hospital for Sick Children, peripheral blood from healthy male donors was drawn in K2-EDTA blood collection tubes (Becton, Dickinson and Co., Franklin Lakes, NJ, USA). Isolation of neutrophils was performed using PolymorphPrep (Axis-Shield, Oslo, Norway), as previously stated following the manufacturer’s guidelines [6,8,9,10,18]. To separate the neutrophils, an equal volume of blood was laid over PolymorphPrep solution and centrifuged at 600× *g*, for 35 min at room temperature without applying acceleration or brakes. After density gradient centrifugation, the polymorphonuclear neutrophil band was transferred to another tube. To eliminate the PolymorphPrep residue, the collected neutrophils were washed by addition of washing buffer (0.425% (*w*/*v*) NaCl with 10 mM HEPES). Furthermore, the red blood cells were lysed twice (as needed) with 0.2% (*w*/*v*) hypotonic NaCl solution for 30 s followed by the addition of an equal volume of 1.6% (*w*/*v*) NaCl solution containing 20 mM HEPES. Finally, two more washes were performed to remove red blood cells debris and soluble components. Neutrophils were resuspended in RPMI medium (Invitrogen) containing 10 mM HEPES (pH 7.2) for further use. A fraction of cells was used for counting, viability test, and quality checks by Haemocytometer, trypan blue method and cytopsin imaging, respectively. Only pure neutrophil preparations with >95–98% viability were used in the experiments. For each experiment, multiple donors were used to get enough biological replicates, as stated in the figure legends. Each experimental data point was obtained from the average of two technical duplicates.

### 2.6. SytoxGreen NETosis Assay

SytoxGreen (Life Technologies), is a cell impermeable DNA binding dye used to assess the NETosis real-time kinetics under different agonist and inhibitors conditions as stated earlier [6,8,9,10,12,19]. A volume of 100 μL media containing 50,000 neutrophils mixed with 5 μM SytoxGreen was seeded into 96-well plates. These neutrophils were activated either with ethanol (vehicle −ve control), lipid fraction-3 (Ft-3), palmitic acid (PA), palmitoleic acid (PO), stearic acid (SA), oleic acid (OA), or F-acid (F6). After cell activation, the fluorescence of the dye was measured at 504 nm excitation and 523 nm emission with the POLARstar OMEGA fluorescence microplate reader (BMG Labtech) at 30-min time intervals for up to 240 min. To calculate the % NETosis in each condition, the green fluorescence at time 0-min was subtracted from the fluorescence at each time point and was then divided by the fluorescence values of cells lysed with 0.5% (*v*/*v*) Triton X-100, at the last time point (representing total DNA). The DNA release in each condition is presented as the percentage of DNA release of total DNA. We ensured that the spontaneous background activation in these samples is less than 30% (of total DNA, by SytoxGreen assay) to minimize variability. The NOX-inhibitor (DPI; diphenyl iodonium) and ATP-uncoupler mitochondrial ROS inhibitor (DNP; 2,4-dinitrophenol) were used with these compounds to test the types of NETosis. Further, the involvement of different MAPKs including ERK, P38, JNK, Akt, and transcriptional firing was monitored by using the specific inhibitors (FR180204, SB202190, SP600125, Akt inhibitor XI, and Actinomycin-D, respectively) during NETosis in neutrophils treated with different fatty acids. Each condition was tested with a technical duplicate. Biological replicates (*n*-values) of independent experiments were reported in figure legends.

### 2.7. Detection of NADPH Oxidase-Mediated Reactive Oxygen Species production

DHR123 (Thermo Fisher Scientific), a reactive oxygen species (ROS) indicator dye was used to measure the intracellular ROS production in neutrophils under different conditions of lipid preparations and PMA. Neutrophils were incubated with DHR123 (20 μM) for 10 min at 37 °C in a CO_2_ incubator as per the manufacturer’s instructions with a brief modification, as we reported earlier [6,9,10,12,20]. DHR123-loaded neutrophils were seeded into 96-well plates (100 μL) and activated with either media only (−ve control), F6, PA, PO, SA, or OA. The fluorescence was measured every 10 min up to 60 min, by an Omega fluorescence microplate reader and ROS generation kinetics were plotted against different conditions.

### 2.8. Mitochondrial ROS Detection

A plate reader assay was performed using the MitoSOX probe, to assess the mitochondrial ROS (mROS) generation under different conditions of NOX-independent agonist, ionomycin and F6, PA, PO, OA, or SA [6,12,19]. Neutrophils (1 × 10^6^ cells per mL) pre-loaded with MitoSox (4 µM), were incubated in incubator at 5% CO_2_ for 15 min at 37 °C. After the incubation, a volume of 100 µL cells were seeded in each well of a black clear bottom 96-well plate and stimulated with F6 or the fatty acids described above. After a 1-h treatment with the lipid fraction and individual compounds, cells were induced for NETosis by ionomycin. Directly following cell activation, the fluorescence of MitoSOX oxidation was measured every 10 min up to 60 min using the SpectraMax Gemini EM fluorescence microplate reader (Molecular Devices, San Jose, CA, USA) at excitation = 510 nm and emission = 580 nm.

### 2.9. Immunofluorescence Confocal Imaging

Chamber slides were used for high-resolution images to verify the release of NETs under different conditions. A volume of 100 μL RPMI with 10 mM HEPES buffer containing 100,000 neutrophils were seeded into 12-well chamber slides (BD Falcon). The neutrophils were activated with media only (−ve control), ionomycin in the presence or absence of Ft-3, F6 or PA, PO, OA, and SA for 120 min at 37 °C in a CO_2_ incubator. The cells and NETs were fixed with paraformaldehyde (4% (*w*/*v*) for 15 min; 2% (*w*/*v*) for overnight) and immunostained with various NET markers as described in earlier reports [6,7,8,9,10]. Briefly, the mouse anti-myeloperoxidase (MPO) antibody (ab25989; Abcam, Cambridge, United Kingdom) at 1:500 dilution was used for staining MPO (with a secondary antibody conjugated with a green fluorescence Alexa flour 488 dye; 1:2000 dilution; Thermo Fisher Scientific), while rabbit anti-citrullinated histone 3 antibody (ab5103; Abcam, Lot # GR273046-3) at 1:500 dilution was used for detecting the presence of citrullinated histone H3 (CitH3, with secondary antibody conjugated with a far-red fluorescence dye Alexa fluor 647; 1:1000 dilution; Thermo Fisher Scientific). To detect histone H3 by immunostaining, a polyclonal rabbit anti-histone H3 antibody (cat#9715; Cell Signaling) at 1:500 was used followed by a secondary antibody conjugated with Alexa fluor 488 dye at 1:1000 dilution (Cat #A11008; Invitrogen). DNA was stained with 4′,6-diamidino-2-phenylindole (DAPI) at 1:1000 dilutions. After treating the secondary antibody, slides were washed and mounted by glass coverslips (Fisher Scientific) with an antifade fluorescent mounting medium (Dako). The images were then taken using an Olympus IX81 inverted fluorescence microscope with a Hamamatsu C9100-13 back-thinned electron multiplying-CCD (EM-CCD) camera and Yokogawa CSU × 1 spinning disk confocal scan head with Spectral Aurora Borealis upgrade, four separate diode-pumped solid-state laser lines (Spectral Applied Research, 405, 491, 561, and 642 nm). The images were taken at 40×/0.95 magnification and processed by Volocity software (version 6.3, Cell Imaging Perkin-Elmer, Waltham, MA, USA) for the image analyses.

### 2.10. Statistical Analysis

Raw data compilation and normalization were calculated in Excel, whereas statistical analysis and graph generation were achieved by using the GraphPad Prism software (Version 5.0a). A student’s t-test for comparing two groups and analysis of variance (ANOVA) with Bonferroni’s post-test or Dunnett’s test for more than two groups were used where appropriate. All data are presented as mean ± standard error from the mean (SEM). A *p*-value of < 0.05 was considered as statistically significant. The biological replicates and applied statistics are mentioned in each of the figure legends.

## 3. Results

### 3.1. Fraction Ft-3 Induces NETosis

**Figure 1:**
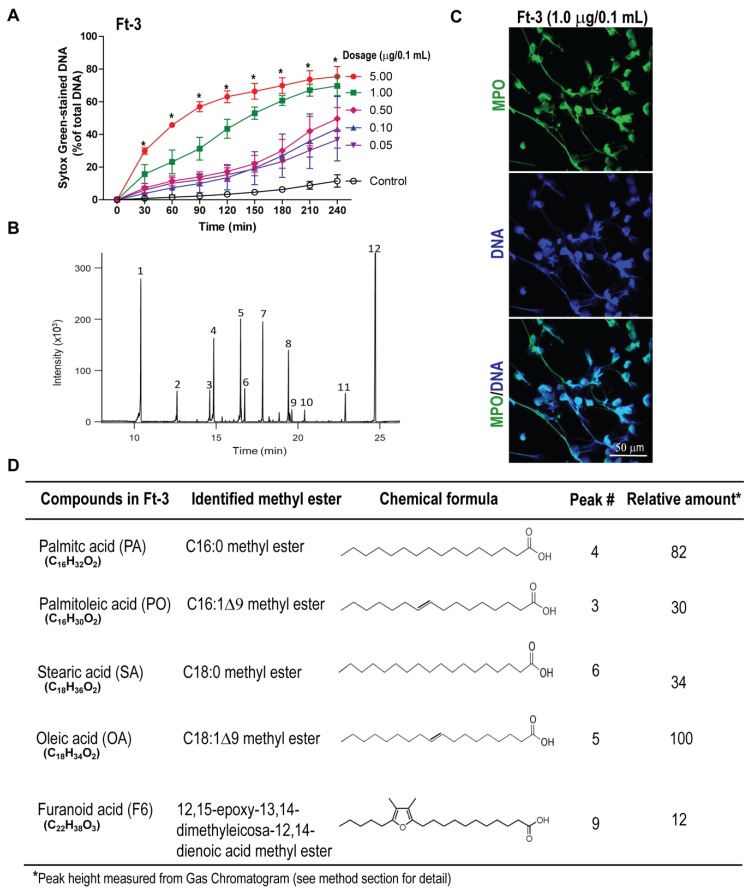
Lipid fraction Ft-3 from total lipid extract of catfish epidermal gel secretion induces neutrophil extracellular trap (NET) formation (NETosis) in human neutrophils. (**A**) SytoxGreen-based NETosis kinetics suggest that lipid fraction 3 (Ft-3), isolated from the dermal gel secretion of the catfish, induces NETosis in a dose-dependent manner (*n* = 5, * *p*-value < 0.05 comparing with control; two-way analysis of variance (ANOVA) with Bonferroni’s multiple comparison post-test); (**B**) Confocal images of neutrophils treated with Ft-3 showing DNA-fibers (blue) colocalized with MPO (green), confirming the formation of NETs by this lipid fraction (*n* = 4; scale bar, 50 µm). See Supplementary Figure S1 for the control NETosis induced by typical agonists phorbol 12-myristate 13-acetate (PMA) and A23187, and confocal images; (**C**) Gas chromatography–mass spectrometry (GC-MS) profile of the methylated fraction present in Ft-3; (**D**) Relative amounts of the studied lipid compounds that were present in Ft-3 fraction (chemical structures shown as the free acids), as determined by GC-MS. See Supplementary Figure S2 for confirmation of the compounds listed in (**D**).

To identify lipids with NETotic ability, we first screened various chromatographic fractions obtained from a total lipid extract of epidermal gel secretion from the Arabian Gulf catfish, using a SytoxGreen plate reader assay. SytoxGreen is a cell impermeable dye that fluoresces green upon binding to DNA released by neutrophils during NETosis [6,8,14]. The fluorescence signals were recorded after incubating human neutrophils with different concentrations of lipids (Fraction #Ft-3) or vehicle control (ethanol only; −ve control) as a proxy for NETosis. The fluorescence data were normalized by the total fluorescence intensity obtained after lysing the neutrophils with Triton-X 100 (100% DNA). These assays identified that the fraction Ft-3 had the potential to induce NETosis in human neutrophils, in a dose-dependent manner (*p* < 0.05; Figure 1A). The dose response kinetics suggest that lipids in Ft-3 may induce both NOX-dependent (e.g., similar to the slow kinetics induced by PMA) and NOX-independent NETosis (similar to the fast kinetics induced by A23187; Supplementary Figure S1). SytoxGreen could also enter cells that has damaged membranes (e.g., necrosis). Therefore, to confirm NETosis, we immunostained the specimen with MPO (green) and stained the DNA with DAPI (blue). Confocal microscopy images (e.g., obtained for Ft-3 fraction with 1.0 μg/0.1 mL lipid; Figure 1B showed colocalization of MPO and DNA confirming that lipids in Ft-3-induced NETosis.

To determine the compounds present in fraction Ft-3, we used GC-MS on the methylated fraction, see Figure 1C. The analysis showed that Ft-3 contained lipid molecules including long-chain fatty acids such as palmitic acid (PA; 16:0), palmitoleic acid (PO; 16:1), stearic acid (SA; 18:0), oleic acid (OA; 18:1), and an F-acid (F6; a 20 carbon-long fatty acid with a furan ring, 5 carbon atoms from the methyl end; Figure 1D). We have confirmed the peaks indicated in Figure 1D using mass-spec detailing the ionization patterns of purified compounds, see Supplementary Figure S2. These long-chain fatty acids are metabolic products of various cells and secretions whereas F-acids are present at high concentrations in certain organs and algae. None of these compounds were known to induce NETosis. Therefore, we conducted detailed studies using the purified lipid compounds.

### 3.2. F6 Is a More Potent NETosis Inducer Than Other Common C16 and C18 Long-Chain Fatty Acids

**Figure 2:**
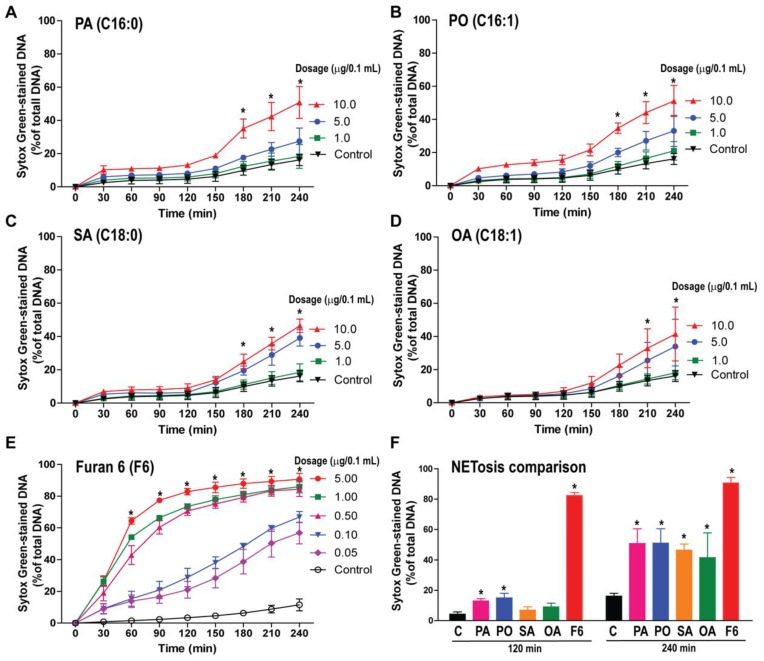
Furanoid acid (F-acid) F6 contributes to most of the rapid NETosis effect exerted by lipid fraction-3 (Ft-3). The SytoxGreen NETosis kinetics analysis was performed in the neutrophils treated either with −ve control (ethanol), or with different concentrations of palmitic acid (PA; 16:0), palmitoleic acid (PO; 16:1), stearic acid (SA; 18:0), oleic acid (OA; 18:1), or furanoid acid (F6). (**A**–**D**) The % DNA release data show slow kinetics and a lower % DNA release in neutrophils activated with PA, PO, SA, or OA. By contrast, F6-treated neutrophils (**E**) showed rapid kinetics and a greater % DNA release (*n* = 3, * *p*-value < 0.05 compared with the control; two-way ANOVA with Bonferroni’s multiple comparison post -test); (**F**) The comparative analyses of the % DNA release between F6 and other fatty acids showed up to 65% more NET release by F6 at 120 and 240 min (*n* = 3, * *p*-value < 0.05; One-way ANOVA with Dunnett’s post-test, compared to control.

To determine the NETotic ability of the lipid compounds of fraction Ft-3, we incubated neutrophils with different concentrations of pure lipid compounds and monitored NETosis by SytoxGreen assays. Common long-chain fatty acids PA, PO, SA, and OA showed dose-dependent but slow NETosis kinetics (*p* < 0.05; Figure 2A–D). The observed kinetics are typical for NOX-dependent NETosis, e.g., Supplementary Figure S1. By contrast, F6 induced a dose-dependent but rapid NETosis particularly at higher doses (*p* < 0.05) see Figure 2E. Rapid kinetics are typical for NOX-independent NETosis, e.g., Supplementary Figure S1. NETosis data at 120 min and 240 min time points, see Figure 2F, highlight the drastic induction of NETosis by F6 compared to the other four long-chain fatty acids.

**Figure 3:**
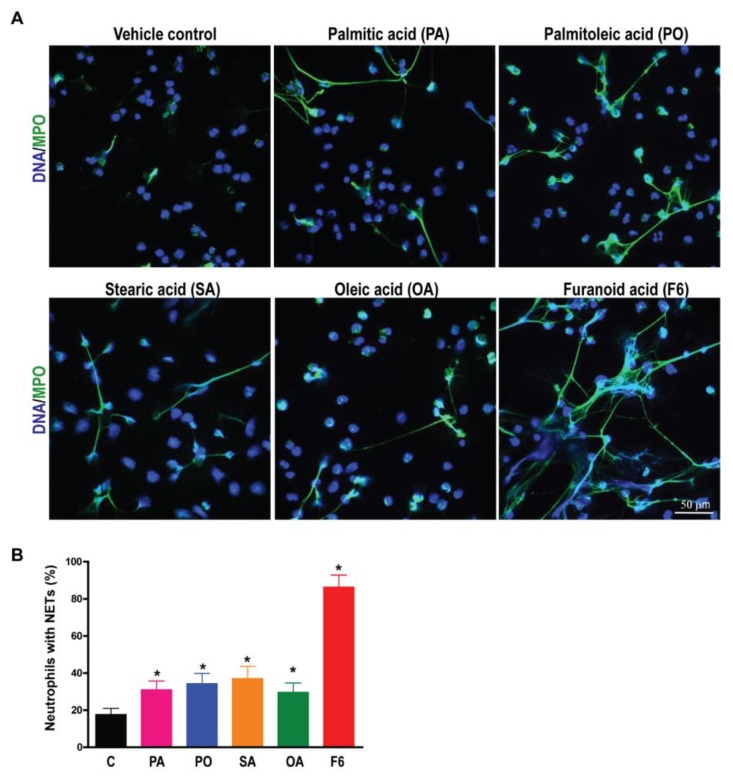
Confocal images confirm that the fatty acids induce NETosis. (**A**) Neutrophils were treated either with −ve control (ethanol), +ve controls (PMA, A23187), 5 μg/0.1 mL of PA, PO, SA, OA, or F6 for 4 h. After 4 h the cells were fixed, immunostained, and imaged for myeloperoxidase (MPO) and DNA. MPO co-localizes to NET-DNA generated by PA, PO, SA, OA, or F6. Images show abundant NET-DNA and MPO colocalization in F6-treated cells, while other fatty acids show a lower amount of NET-DNA staining, confirming the SytoxGreen kinetics data (Blue, DAPI (4′,6-diamidino-2-phenylindole) staining for DNA; green, MPO; *n* = 3; scale bar 50 μm). See Supplementary Figures S1 and S2 for −ve control and +ve controls treated with PMA and A23187 images, and single channel images; (**B**) Neutrophils with NETs were quantified (*n* = 3; *, *p* < 0.05 compared to control; One-way ANOVA followed by the Dunnett’s post-test).

To confirm whether these fatty acids induce NETosis, specimens were visualized by immunofluorescence confocal microscopy. Neutrophils treated with F6 or PA, PO, SA, and OA were fixed at 240 min post-stimulation and stained for DNA with DAPI (4′,6-diamidino-2-phenylindole) and immunostained for MPO with fluorescently labeled antibodies. Confocal images showed that MPO (green) decorated the DNA (DAPI, blue), both within the decondensing nuclei and on the extracellular NET structures, see Figure 3A,B and Supplementary Figure S1. Neutrophils treated with an ethanol-only vehicle (−ve control; up to 0.6% (*v*/*v*) ethanol based on the volume used in resuspending the lipid compounds) showed intact nuclei and a few neutrophils with NETs. Therefore, F6 or PA, PO, SA, and OA induce NETosis. Although all of these compounds could induce NETosis, F6 is a potent and fast inducer of NETosis, see Figure 2 and Figure 3.

### 3.3. F6 Induces the Production of Both NOX- and Mitochondria-mediated ROS, Whereas Other Long-Chain Fatty Acids Induce Only the Production of NOX-mediated ROS

**Figure 4:**
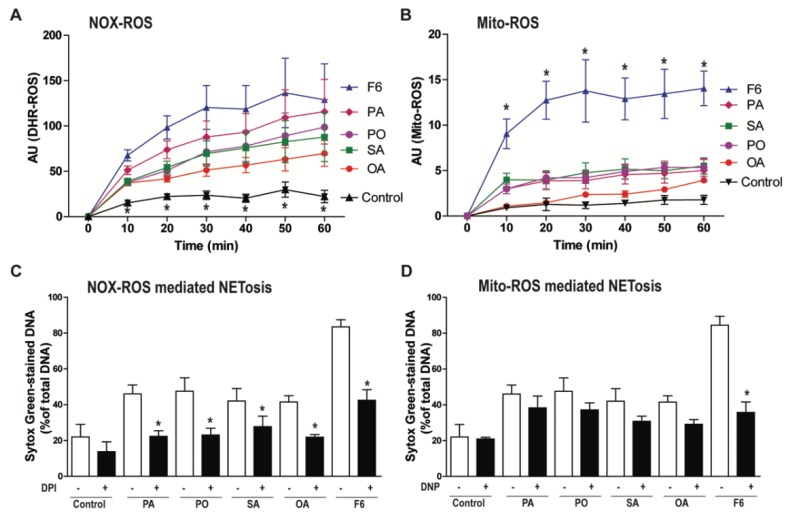
F6 modulates NETosis through both a NOX-dependent and NOX-independent pathway. (**A**) To confirm the involvement of NOX-mediated ROS production, a DHR123 assay was performed. Neutrophils were treated with cytosolic ROS indicator dye DHR123 and activated either with ethanol (−ve control), 5 μg/0.1 mL of PA, PO, SA, OA, or F6. ROS generation was assessed over 60 min post activation by a plate reader; (**B**) To examine the generation of mitochondrial ROS, MitoSox dye was used during the plate reader assay. Neutrophils were treated with MitoSox, a mitochondrial ROS indicative dye for 15 min, and activated either by ethanol (−ve control), 5 μg/0.1 mL of PA, PO, SA, OA, or F6 and the kinetics were assessed (*n* = 3, * *p* < 0.05 comparing with control; two-way ANOVA with Bonferroni’s multiple comparison post -test); (**C**,**D**) To test the involvement of the NETosis pathway, neutrophils were pre-incubated either with NOX-inhibitor (DPI; 1 µM) or mitochondrial ROS inhibitor (DNP; 25 µM) for 1 h, followed by activation either with ethanol (−ve control), 5 μg/0.1 ml of PA, PO, SA, OA, or F6. The % DNA release data at 4 h of activation, show NETosis suppression by diphenyl iodonium (DPI) in neutrophils activated with PA, PO, SA, OA, or F6 (5 µg/0.1 mL), while (**D**) 2,4-dinitrophenol (DNP) (ATP uncoupler; mitochondrial ROS inhibitor) only suppresses the NETosis induced by F6 (*n* = 4, * *p* < 0.05 comparing between compound with and without inhibitors; independent *t*-test).

NETosis requires reactive oxygen species (ROS) production either by NOX or mitochondria [9,10,18]. Therefore, we investigated the involvement of NOX-ROS and mitochondrial ROS (Mito-ROS) during the induction of NETosis by F6, PA, PO, SA, or OA. NOX ROS is detectable by a DHR123 assay [20]. When ROS oxidizes DHR123, it becomes R123, which fluoresces green. DHR123 plate reader assays showed that all of these fatty acids induced the production of higher amounts of ROS compared to the negative control (ethanol-only vehicle; *p* < 0.05; Figure 4A). Interestingly, a substantial amount of mitochondrial ROS generation (detected by mitochondrial-specific MitoSox) was only seen in the neutrophils treated with F6, but not PA, PO, SA, and OA, as shown in Figure 4B. This dataset indicates that F6 could induce the generation of ROS from both NOX and mitochondria, but other long-chain fatty acids induce ROS only from the NOX pathway.

To further verify the source of ROS generation, we used pathway-specific inhibitors (diphenyleneiodonium, DPI is a NOX-inhibitor; 2,4-dinitrophenol, DNP is a mitochondrial uncoupler) in the NETosis assays. Neutrophils were pre-treated with vehicle, DPI, or DNP for 1 h and then the compounds (either F6, PA, PO, SA, or OA) were added to assess the NETosis by SytoxGreen assays. DNA release data at the last time point (240 min) showed that NOX-inhibitor (DPI) suppressed NETosis induced by all the lipid agonists, as shown in Figure 4C. By contrast, mitochondrial ROS inhibitor (DNP) only suppressed NETosis induced by F6, but not other long-chain fatty acids, see Figure 4D. This data confirms that F6 induces NETosis through both NOX-dependent and NOX-independent pathways whereas PA, PO, SA, and OA only induce NOX-dependent NETosis, see Figure 4. Therefore, the involvement of both pathways of NETosis induced by F6 compared with PA, PO, SA, and OA could be responsible for the rapid and efficient NETosis induced by F6 compared to PA, PO, SA, and OA.

### 3.4. F6, but Not Other Long-Chain Fatty Acids Induce Citrullination of Histone H3 (CitH3)

**Figure 5:**
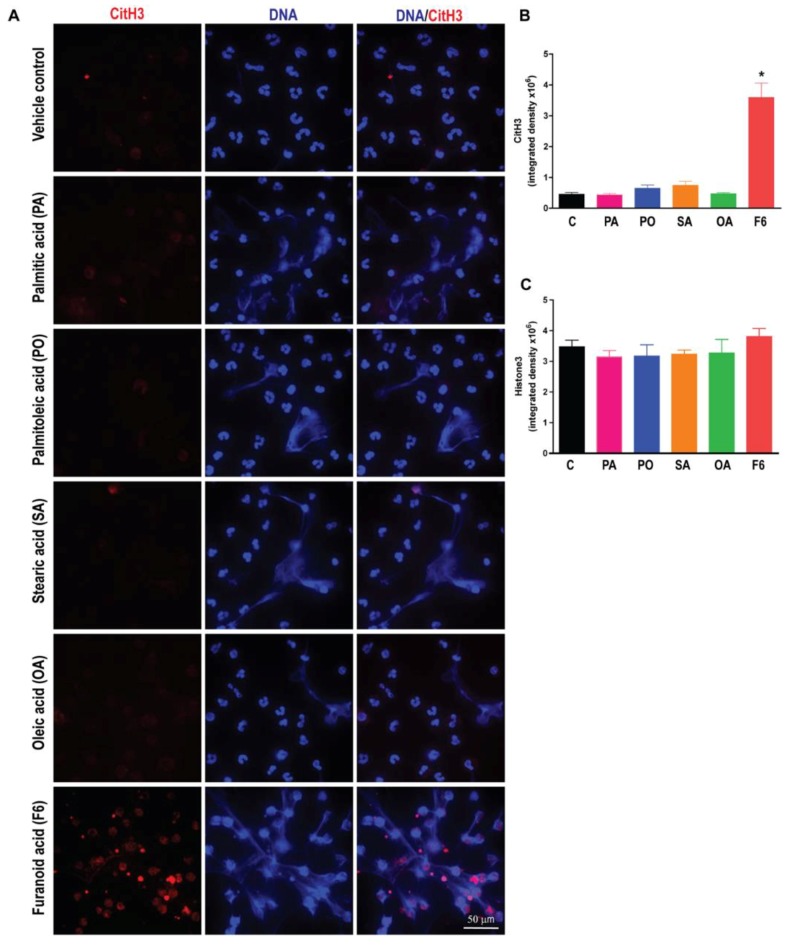
F6 induces histone H3 citrullination (CitH3) during NETosis. (**A**) Neutrophils were incubated either with ethanol (−ve control), A23187 (+ve control), 5 μg/0.1 mL of PA, PO, SA, OA, or F6 for 120 min followed by the fixation and immunostaining of the CitH3. Cells were stained for CitH3 (red) and DNA (DAPI; blue). Confocal fluorescence images show the substantial CitH3 immunostaining in neutrophils treated with F6, while the control and PA-, PO-, SA-, or OA-treated neutrophils lack CitH3 immunostaining (scale bar, 50 μm; images are representative of three independent experiments). See the Supplementary Figure S4 for the CitH3 in the positive control A23187-treated neutrophils; (**B**) CitH3 was quantified from the images using Image J (*n* = 3; * *p* < 0.05 compared to control; One-way ANOVA followed by Dunnett’s post-test); (**C**) Histone H3 was quantified from the images using Image J (*n* = 3; One-way ANOVA; the differences in these controls are not significantly different; images not shown).

Citrullination of histone 3 is a hallmark of NOX-independent NETosis [6,12,16,21]. Therefore, we examined the CitH3 in neutrophils treated with all the fatty acids (F6, PA, PO, SA, or OA). The confocal immunofluorescence microscopy detected obvious CitH3 formation in F6-treated but not in PA-, PO-, SA-, or OA-treated neutrophils, see Figure 5. Specificity of CitH3 formation in the NOX-independent pathway was verified by A23187, which is an efficient inducer of CitH3 formation in neutrophils, see Supplementary, Figure S4. NETosis could be quantified and confirmed by co-localization of various NET markers (e.g., MPO, elastase, DNA) or by examining CitH3 and baseline levels of histone H3 5. These data show that F6, but not C16 and C18 fatty acids effectively induces citrullination of histone during NETosis.

### 3.5. F6 and Long-Chain Fatty Acids Activate Different Sets of Kinases

**Figure 6:**
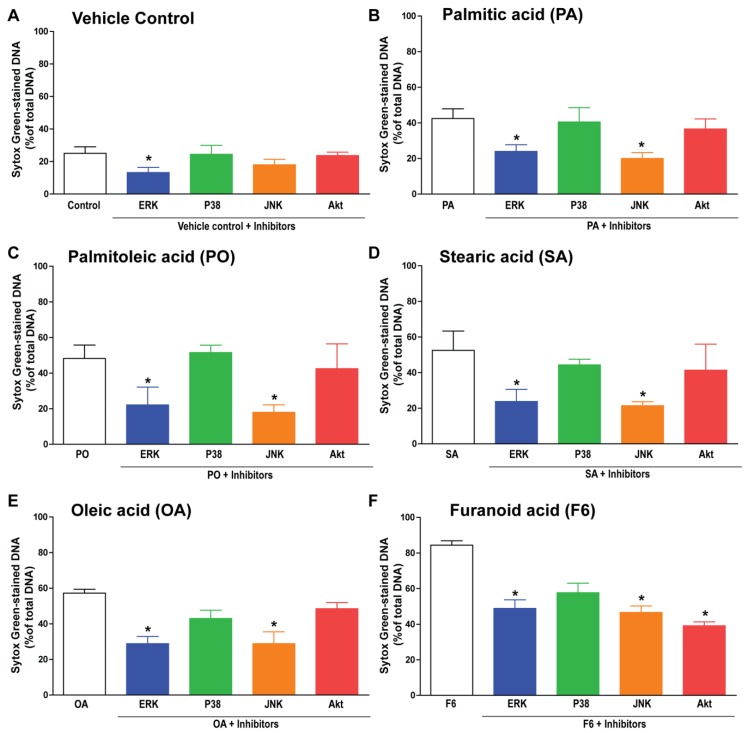
Different sets of kinases are involved in PA-, PO-, SA-, OA-, or F6-mediated NETosis. Neutrophils were pre-incubated for 1 h either with different kinase inhibitors Akt inhibitor XI, FR180204, SB202190, SP600125 for Akt, ERK, P38, and JNK, respectively, followed by incubation with ethanol (−ve control), PA, PO, SA, OA, or F6. (**A**–**E**) The % DNA release data at the final time point (240 min) show background NETosis suppressed by ERK inhibitor while ERK and JNK inhibitors suppress the NETosis induced by PA, PO, SA or OA; (**F**) By contrast, F6-mediated NETosis was suppressed by Akt, ERK, and JNK inhibitors (*n* = 3, * *p* < 0.05 comparing between compound with and without inhibitors; One-way ANOVA with Dunnett’s post-test).

Different sets of kinases are responsible for inducing NOX-dependent and NOX-independent NETosis [6,8]. Therefore, we examined the importance of MAPKs (ERK, p38, and JNK) and another kinase Akt during fatty acid-induced NETosis. NETosis inhibition at the final time point (240 min) indicated the involvement of ERK in baseline activation of NETosis whereas ERK and JNK were important for the induction of NETosis by the fatty acids, F6, PA, PO, SA, or OA; Figure 6A–F. Akt was also important for the F6-mediated induction of NETosis, see Figure 6F. These data indicate the involvement of different sets of kinases during the activation of NETosis by F6 and C16 and C18 long-chain fatty acids.

### 3.6. NETosis by the Compounds Involves Transcription

**Figure 7:**
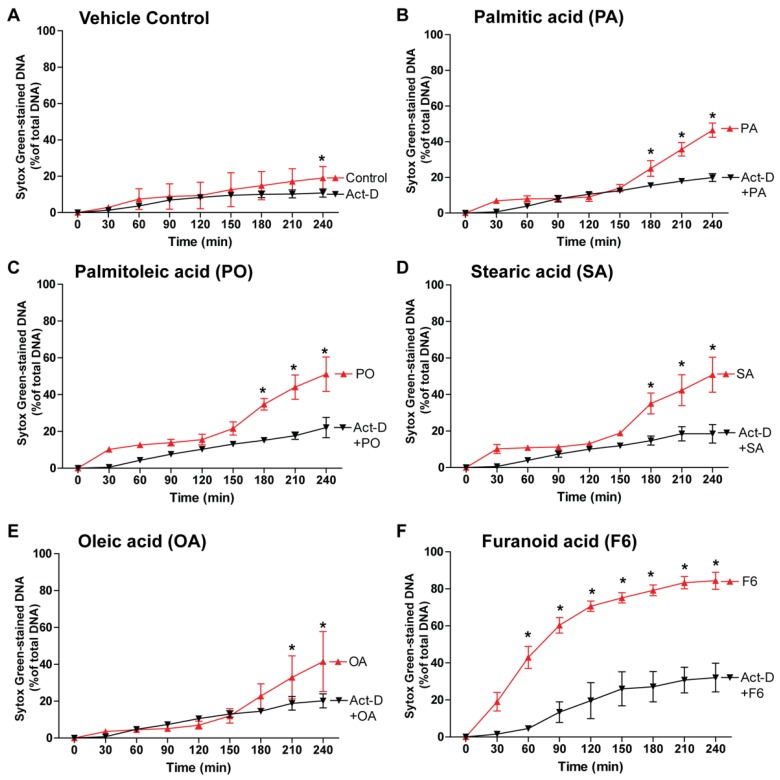
Fatty acid-induced NETosis involves transcriptional firing. (**A**–**F**) Neutrophils were pre-incubated 1 h either with actinomycin-D followed by incubation with ethanol (−ve control), PA, PO, SA, OA, or F6. NETosis kinetics data indicate that the transcriptional firing is important for all the tested compounds as actinomycin-D suppresses NETosis released by these fatty acids (*n* = 3–4, * *p*-value < 0.05 comparing between compound with and without inhibitors; two-way ANOVA with Bonferroni’s multiple comparison post-test).

The activation of different kinase cascades leads to the transcriptional firing and chromatin decondensation in both NOX-dependent and NOX-independent NETosis [8]. Therefore, to determine whether fatty-acid-mediated NETosis also involves transcription, we induced NETosis using the fatty acids, F6, PA, PO, SA, or OA, in the presence or absence of the transcription inhibitor, actinomycin D (Act-D; Figure 7A–F). Since Act-D suppressed the NETosis induced by these agonists, all the fatty acids that go through the transcription-mediated chromatin decondensation induce NETosis.

## 4. Discussion

Over the past several years, one of us (JMAH) has demonstrated that preparations from the gel secreted from the skin of the catfish, found in the waters of the Gulf region have wound healing properties in humans [22,23,24]. These studies used preparations that contained proteins and lipids. In this report, we focused on determining NETosis-related properties of the lipid components of the gel. Among the partially purified lipid fractions, Ft-3 contained components with NETosis-inducing activity. We demonstrate herein that a minor component F6 (an F-acid) of the fraction Ft-3 induces a high degree of NETotic activity. Our finding that F6 induces both NOX-dependent and NOX-independent NETosis is noteworthy. Effect of F6 on NETosis is similar to hepoxilins [17]. Some of the remaining activity is ascribable to the long-chain fatty acids (PA, PO, SA, and OA) that induce NETosis via a NOX-dependent pathway. Furthermore, NETosis induced by these lipids involves the key MAPKs and transcriptional firing. These mechanistic insights are useful for designing appropriate lipid formulations to regulate inflammation-related diseases involving NETosis.

Palmitic acid (PA; C16:0), palmitoleic acid (PO; C16:1), stearic acid (SA; C18:0), and oleic acid (OA; C18:1) are some of the common long-chain fatty acids present in the cells and tissues [25,26,27,28,29,30]. However, neither these long-chain fatty acids nor F6 have been reported to induce NETosis. Our studies show that saturated and unsaturated C16 and C18 fatty acids are capable of inducing NETosis via a NOX-dependent pathway. Since these fatty acids are present in high concentrations in the Ft-3 fraction, these lipids could contribute to the NETosis induced by the lipid mixture. Nevertheless, the requirement of higher concentrations to induce NETosis may limit the therapeutic potential of C16 and C18 fatty acids. Concentrations of these fatty acids could be high in specific tissues and secretions due to cellular metabolism and inflammation [31,32,33]. Therefore, these compounds may regulate NETosis in vivo.

Wakimoto et al. have demonstrated the presence of F-acids in the green lipped mussel *Perna canaliculus* and showed them to possess anti-inflammatory actions in a rodent model of adjuvant-induced arthritis [34]. F-acid could affect multiple cells and systems to exert its overall function in vivo. Therefore, understanding the effect of F6 on specific cells is important to delineate specific mechanisms. F6 has not previously been studied in the context of NETosis. We show that F6 is potent in inducing NETosis, more so than other long-chain fatty acids present in the same lipid fraction Ft-3, and its effect is mediated through both NOX-dependent and NOX-independent pathways. Furanoids are believed to have anti-inflammatory properties, perhaps through their antioxidant properties [34,35,36,37,38,39]. Our studies imply that F6-mediated induction of NETosis may help to effectively compact infection and to resolve inflammation in vivo.

Data obtained from both F6 and long-chain fatty acids further show that kinase activation is involved in these NETosis. Studies conducted in our lab [6,9] and other labs [40,41,42] shows that ERK and JNK activation occurs in NOX-dependent NETosis. Activation of specific kinases however is dependent on the agonist [8,42,43,44]. Our inhibitor studies suggest that NOX-dependent NETosis induced by all four long-chain fatty acids (PA, PO, SA, and OA) involve ERK and JNK but not Akt. Our recent studies show that lipopolysaccharides (LPS) engagement of TLR4 results in JNK-mediated ROS production and subsequent NOX-dependent NETosis [9]. The receptor for F6 has not yet been clearly established. Since PA is a ligand for TLR4 [45,46], PA and perhaps other fatty acids engage TLR4 to induce NOX-dependent NETosis. Akt activation is considered to be important for NOX-independent NETosis and the autophagy component involved in NOX-dependent NETosis induced by certain agonists [7,47]. Since Akt activation is not essential for long-chain fatty acid-mediated NETosis, autophagy-related pathways may not be involved in the NOX-dependent NETosis induced by these agonists. By contrast, F6-induced NETosis involve the activation of ERK, JNK, and Akt. Involvement of these kinases is consistent with the activation of both NOX-dependent and NOX-independent NETosis by F6.

Activation of various kinase cascades leads to transcription activation [8]. Our previous studies and a few other studies suggest that transcription, but not new protein synthesis by translation, is essential for NETosis [48,49,50]. We have recently proposed via a unifying model that both NOX-dependent and NOX-independent NETosis pathways culminate at the chromatin decondensation step that is regulated by transcriptional firing [8]. This step is also necessary for other NETosis-related cell deaths such as ApoNETosis [19]. C16-, C18-, and F6-induced NETosis also requires the transcriptional step necessary for NETosis, indicating that fatty acids also use similar steps for inducing NETosis.

Several years ago, we embarked on a study to investigate the nature of the wound healing/anti-inflammatory property in human foot ulcers that were unresponsive to conventional treatment and demonstrated in an in vitro study the presence of several factors that may be responsible for this effect. An interesting observation was recorded during the treatment of these ulcers; ulcer healing progressed without the use of antibiotics, although the ulcers were infected by different types of microorganisms [22,23,24]. This observation suggested the presence of antibiotic-like activities. We propose that F6 and other long-chain fatty acids are responsible for some of the therapeutic effects of the total catfish epidermal gel (e.g., wound healing). It is possible that F6 and other lipidic compounds participate with proteins (growth factors identified) present in the total gel to control various aspects of inflammation.

## Supplementary Materials

The following are available online at http://www.mdpi.com/2218-273X/8/4/144/s1, Figure S1: (A) The kinetics of the typical NOX-dependent and -independent NETosis induced by agonists PMA and A23187, respectively. (B) Confocal microscopy images of the immunostained neutrophils activated either with vehicle (-ve control), PMA or A23187 (240-min time point; Blue, DNA (DAPI); Green, MPO; Scale bar, 50 μm); Figure S2: Comparison of mass spectra from compounds in fraction Ft-3 after methyl ester/TMSi derivatization showing mass spectra of authentic standards published in the NIST library (top panels) and the corresponding compounds identified in the catfish fraction Ft-3 (lower panels) investigated in this study. Figure S3: Single chanle representation of the MPO and DAPI stained confocal images taken from Figure 3.; Figure S4: (A) CitH3-immunostained confocal images of the neutrophils treated with either vehicle (ethanol, -ve control) or A23187 (a +ve control) for 120 min (Blue, DNA stained with DAPI; red, CitH3; Scale bar, 50 µm.(B) Integrated density of the CitH3 in multiple images were analyzed by using ImageJ software.
