# Supplementary material for: Furanoid F-Acid F6 Uniquely Induces NETosis Compared to C16 and C18 Fatty Acids in Human Neutrophils
Source: Biomolecules. 2018 Nov 13;8(4):144. doi: 10.3390/biom8040144 (PMC6315434; doi:10.3390/biom8040144)
Supplement: Supplementary file 1 [file biomolecules-08-00144-s001.pdf]

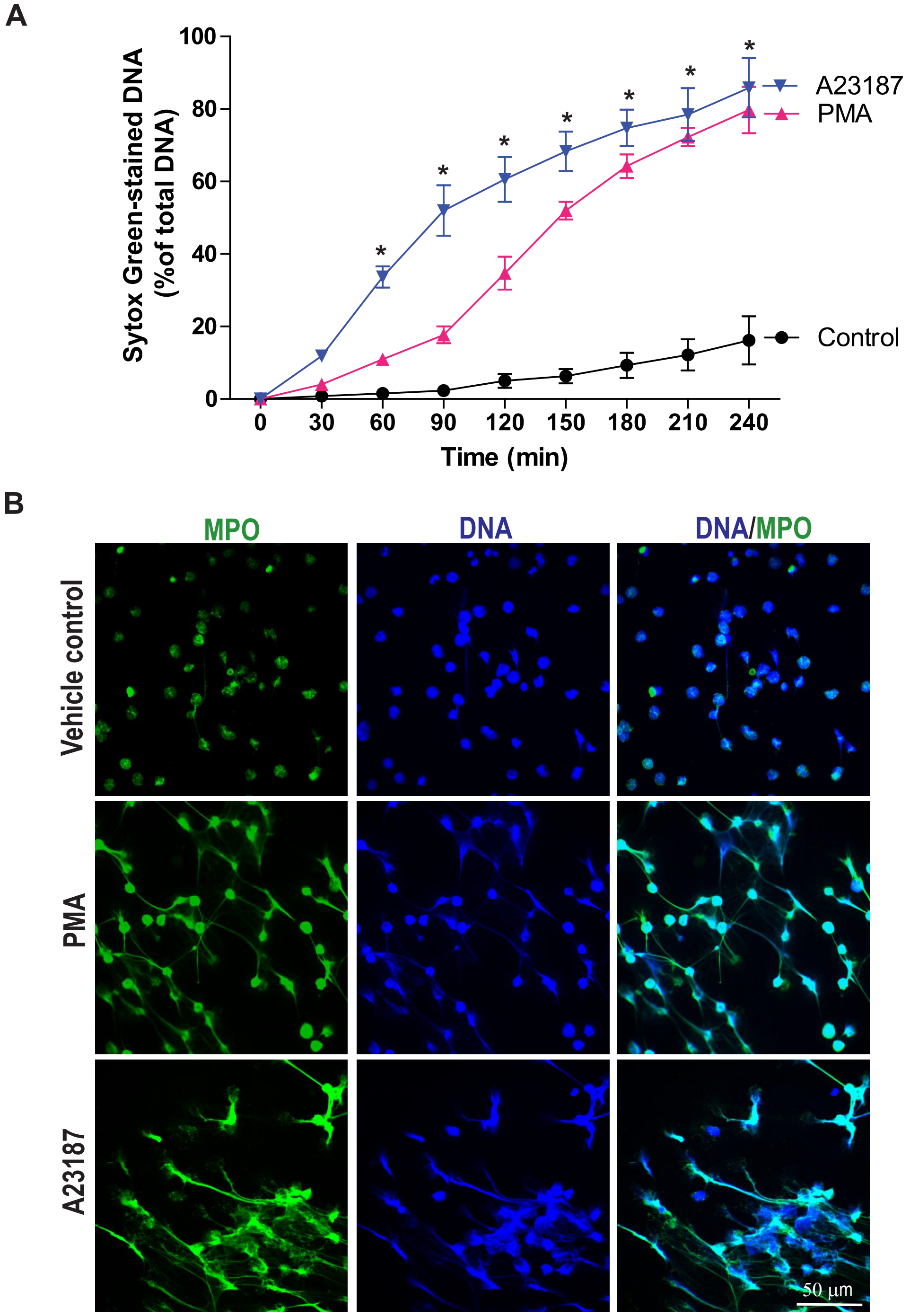

Figure S1. (A) The kinetics of the typical NOX-dependent and -independent NETosis induced by agonists PMA and A23187, respectively. **(B)** Confocal microscopy images of the immunostained neutrophils activated either with vehicle (-ve control), PMA or A23187 (240-min time point; Blue, DNA (DAPI); Green, MPO; Scale bar, 50 μm).

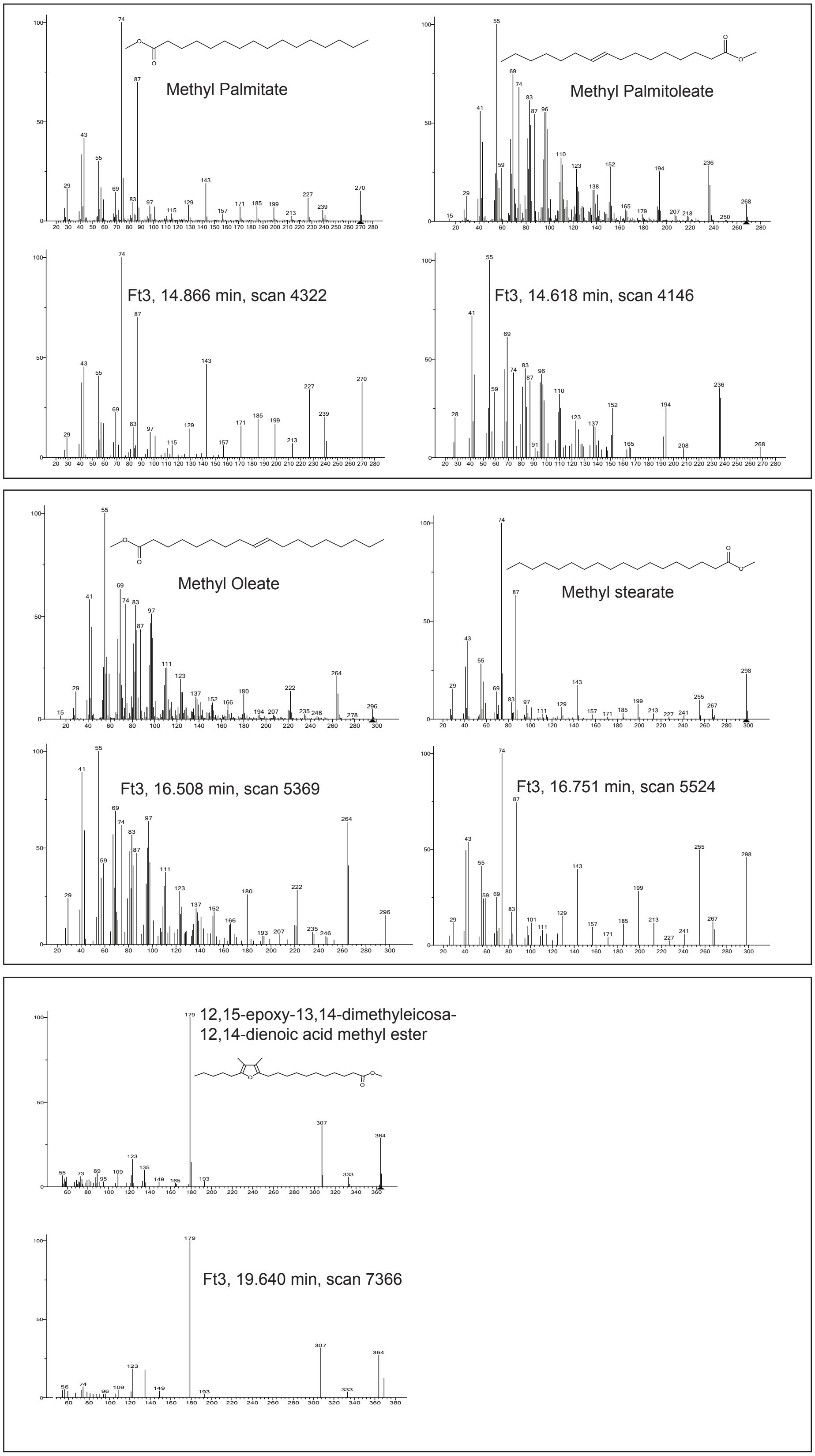

Figure S2. Comparison of mass spectra from compounds in fraction Ft-3 after methyl ester/TMSi derivatization showing mass spectra of authentic standards published in the NIST library (top panels) and the corresponding compounds identified in the catfish fraction Ft-3 (lower panels) investigated in this study.

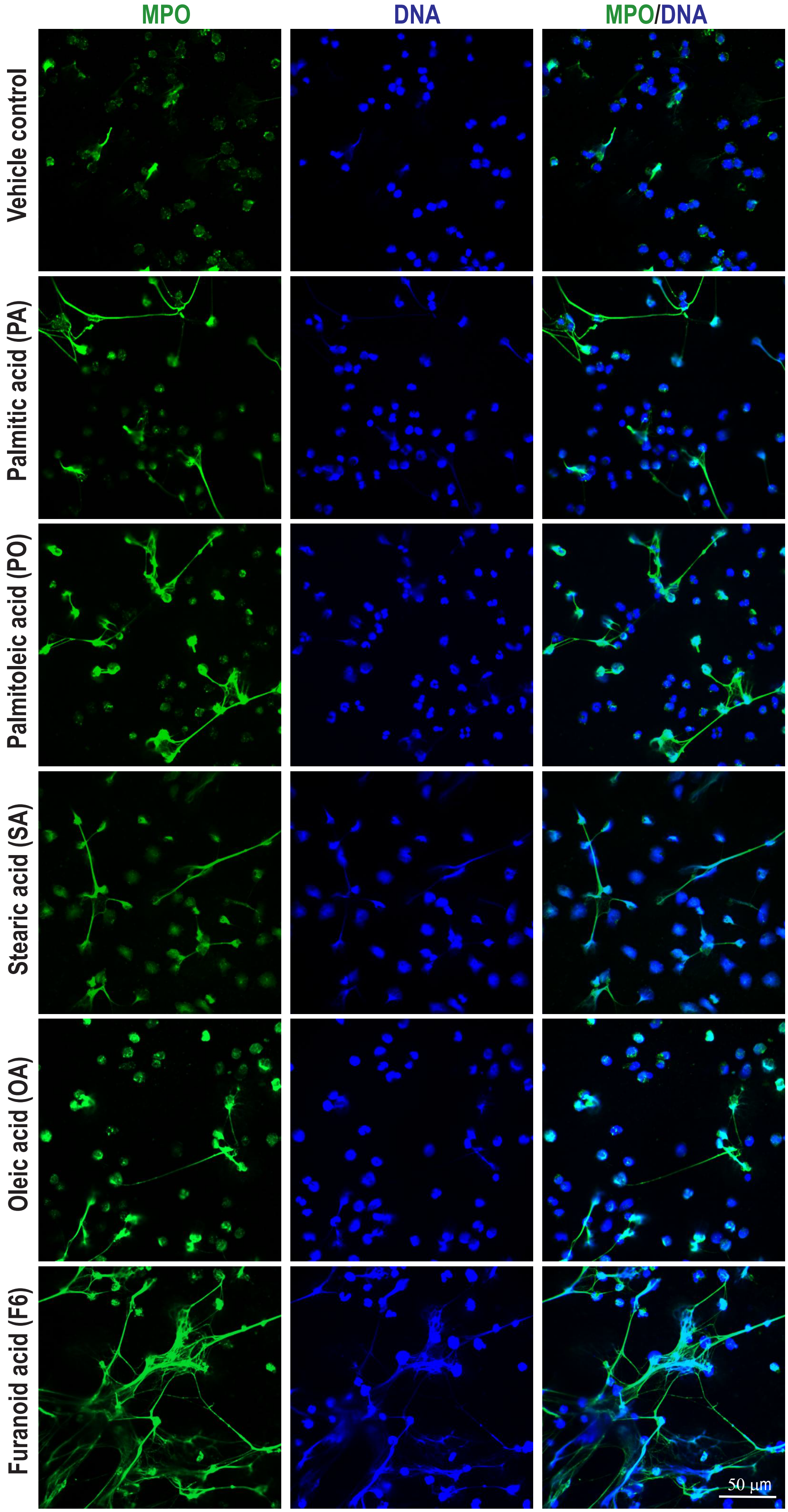

Figure S3. Single chanle representation of the MPO and DAPI stained confocal images taken from Figure 3.

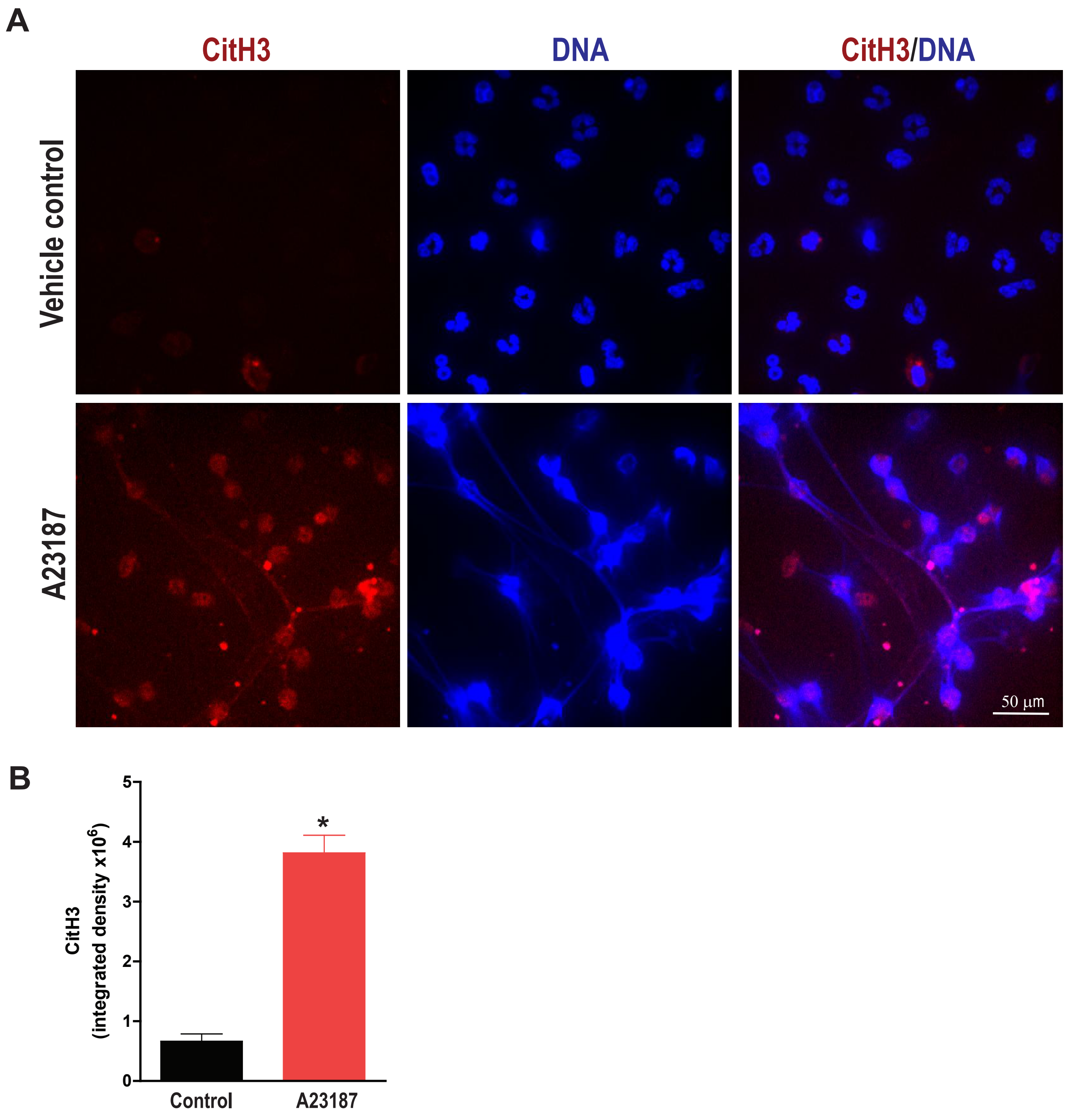

Figure S4. (A) CitH3-immunostained confocal images of the neutrophils treated with either vehicle (ethanol, -ve control) or A23187 (a +ve control) for 120 min (Blue, DNA stained with DAPI; red, CitH3; Scale bar, 50 μm). **(B)** Integrated density of the CitH3 in multiple images were analyzed by using ImageJ software.
